# Discovery of indole tetrafluorophenoxymethylketone-based potent novel small molecule inhibitors of caspase-3

**DOI:** 10.1186/2191-2858-2-27

**Published:** 2012-07-16

**Authors:** Dodheri Syed Samiulla, Andra Naidu, Gummadi Venkateshwar Rao, Murali Ramachandra

**Affiliations:** 1 DMPK Department, Aurigene Discovery Technologies Limited, Electronic City Phase 2, Hosur Road, Bangalore, 560100, India; 2 Department of Chemistry, Jawaharlal Nehru Technological University Hyderabad, Kukatpally, Hyderabad, 500085, India

**Keywords:** Caspase-3, Small molecule, Apoptosis, Inhibition

## Abstract

**Background:**

Caspase-3 inhibition has been demonstrated to be therapeutically effective in moderating excessive programmed cell death. Interest in caspase-3 as a therapeutic target has led many to pursue the development of inhibitors. To date, only a few series of non-peptide inhibitors have been described, and these have limitations on their drug-like properties.

**Methods:**

Here, we report the screening of 70 novel small molecules against the caspase-3 enzyme which belongs to four different series (indole fluoromethylketone, indole difluoro and tetrafluorophenoxymethylketone, and oxalamide). Selected molecules were subjected for counter-screening, cell-based, ADME/PK assays in order to understand the potency and drug-like properties.

**Results:**

The screening yielded series of hits with IC_50_ values ranging from 0.11 to 10 μM with reasonable SAR, irreversible mode of inhibition, and reasonable selectivity against other proteases including caspase-1, cathepsin B and D, and thrombin. On the basis of *in vitro* profile, the selected molecules were evaluated for their drug-like properties. Among the compounds evaluated, compound **3D** exhibited good solubility, low permeability, interaction with efflux pump, and low potential for CYP450 drug-drug interaction. After intravenous administration, compound ***3D*** showed low clearance (588 ml/hr/kg), medium volume of distribution, and good oral bioavailability (90%).

**Conclusions:**

These results support further advancement of compound **3D** in different apoptotic models to develop as a new anti-apoptotic agent in relevant disease conditions.

## Background

Apoptosis or programmed cell death is a critical cellular process in normal development and homeostasis of multicellular organisms. Cells undergo apoptosis in response to a variety of stimuli, and during apoptosis, they do so in a controlled, regulated fashion [1]. A major biochemical pathway involved in the apoptosis includes a family of proteases, known as caspases, which act in a cascade to activate downstream caspases responsible for breakdown or cleavage of key cellular substrates required for normal cellular fashion, including structural proteins in the cytoskeleton and nuclear proteins such as DNA repair enzymes [2,3].

Caspases are found practically in all organisms from *Caenorhabditis elegans* to humans. At least 12 of the caspases have been identified (caspases 1 through 10, 13, and 14). Caspases share similarities in amino acid sequence, structure, and substrate specificity, and are subdivided in to two subfamilies based on their functionality: caspases involved in inflammation (caspases 1, 4, 5, 11, 12, 13, and 14) and apoptosis-related caspases (caspases 2, 3, 6, 7, 8, 9, and 10). Among the identified caspases, activation of caspase-3 is a key event integrating upstream signals into final execution of cell death [4].

**Figure 1:**
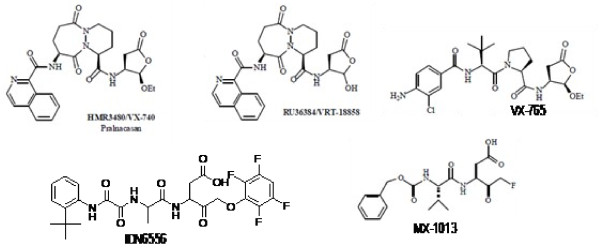
Structures of caspase-3 inhibitors in discovery and development stage.

Abnormally high amounts of apoptosis have been reported in several liver diseases, including alcoholic hepatitis, transplantation, Wilson's disease, and viral hepatitis [5,6]. Several reports demonstrated that inhibition of caspases protect the liver from apoptosis-associated liver injury in preclinical models. Prototypical caspase inhibitors such as ZVAD-FMK have been shown to be efficacious in many animal models, including α-Fas- and TNF-mediated liver injury [7]. More recently, other caspase inhibitors have been shown to be efficacious in rodent models of liver disease [6] (Figure 1). Efficacy with the broad-spectrum caspase inhibitors in preclinical models suggests that they have potential for the treatment of liver diseases in humans. In addition, procaspase-3 concentration is elevated in certain neuroblastomas, lymphomas, leukemias, melanomas, and liver cancer [4]. This makes caspase-3 an interesting therapeutic target, and the search for caspase-3 inhibitors has been an ongoing endeavor by many pharmaceutical companies.

The objective of the present study is to characterize novel small molecule caspase-3 inhibitors with an emphasis on understanding absorption, distribution, metabolism, and excretion (*in vitro* ADME), and *in vivo* pharmacokinetic properties towards achieving desired pharmacodynamic effects and efficacy in preclinical *in vivo* models.

## Methods

### Reagents

Caspase-3 (C1224), *n*-Acetyl-Asp-Glu-Val-Asp-7-amido-4-methylcoumarin (A1086), cathepsin B (C0150), Boc-Gln-Ala-Arg-7-amido-4-methylcoumarin hydrochloride (B4153), *N*-Acetyl-Tyr-Val-Ala-Asp-7-amido-4-methylcoumarin (A2452), *N*-Acetyl-Tyr-Val-Lys (biotinyl)-Asp 2,6-dimethylbenzoyl-oxymethyl ketone (A4339) were procured from Sigma-Aldrich (St. Louis, MO). Caspase-1 enzyme (BML-AK701) was from Enzo Life Sciences, Inc. (NY, USA). Thrombin enzyme (HT1002a) was procured from Enzyme Research Laboratories (IN, USA). Cathepsin B substrate (Z-Phe-Arg-AMC HCl) was from Bachem AG (Bubendorf, Switzerland) (cat. no.: I-1160). Cathepsin inhibitor II-ALLN (cat. no.: 219426) was from Calbiochem AG (EMD Millipore Corporation, MA, USA). All other reagents were of research grade. IDN6556 was synthesized at Aurigene Discovery Technologies Ltd. (Karnataka, India). Selectivity against caspase-7, 8, and 9, and cathepsin-D were performed using Biomol kit (Enzo Life Sciences, Inc.).

### General analytical methods

Fluorescence measurements were done using VICTOR2V 96/384 multilabel plate reader (PerkinElmer Life Sciences, MA, USA) at *λ*_ex_ = 360 nm and *λ*_em_ = 460 nm top readout (ex, excitation; em, emission) in Corning black 96-well flat bottom plates. The enzyme activity was measured at 30°C after 30 min. Trans-epithelial resistance (TEER) measurements (for monolayer integrity) were made using instrument from World Precision Instrument (Florida, USA). HPLC (1100 series, Agilent Technologies Inc., CA, USA) was performed on reverse phase column (Zorbax Eclipse XDB C18, 150 × 4.6 mm, 5 μm). LC-MS/MS was performed in multiple reaction monitoring mode using Applied Biosystems API 3200 (Chromos, Singapore) coupled to Agilent Technologies 1100 series HPLC on a reverse phase column (Zorbax Eclipse XDB C18, 50 × 4.6 mm, 5 μm).

### Compound selection and screening

Focused diversity approach was employed for the synthesis of compounds at Aurigene Discovery Technologies Ltd., and the detailed chemistry has been reported earlier [8,9]. As the active site of caspase-3 contains a sulfhydryl moiety necessary for its catalytic activity, we focused on molecules with the warheads (electrophilic moiety: carbonyl group of fluoromethylketone) capable of reacting with the cysteinyl thiols. We selected 70 novel small molecules (NCEs) with sufficient chemical diversity belonging to four different series (indole fluoromethylketone, indole difluoro and tetrafluorophenoxymethylketone and oxalamide) and purity more than 95%.

Screening of compounds was carried out at two different concentrations (1 and 10 μM). Selected compounds were subjected to dose response determination in semi-log concentration (serial threefold dilutions) at constant DMSO concentration of 1% with highest compound concentration at 100 μM. Counterscreening against some of proteases, permeability, and CYP450 inhibition studies was carried out for few selected compounds at 10 μM concentration.

### Protease inhibition assays

The ability of NCEs to inhibit the activity of human recombinant caspases as well as other proteases was determined using a standard fluorometric microplate assay as described in [1,10]. The assay buffer contained 25 mM sodium HEPES, 50 mM KCl, 0.1% CHAPS, and 1 mM β-mercaptoethanol, pH7.4. To a reaction mixture (100 μL) containing the above buffer, 10 μM AC-DEVD-AMC and 10 U caspase-3 were added in a black 96-well microplate. Activities of the other caspases were determined similarly using the corresponding fluorogenic substrates: 50 U of caspase-1 and 10 μM N-acetyl-Tyr-Val-Ala-Asp-7-amido-4-methylcoumarin.

Thrombin assay reaction mixture (100 μl) contained 140 ng of enzyme and 100 μM of substrate (Boc-Glu-Ala-Arg-AMC) in Tris–HCl buffer pH 8.5. Cathepsin-B assay buffer contained 50 mM MES, 0.01% CHAPS, 100 μM of substrate (Z-Phe-Arg-AMC HCl), and 0.1 U of enzyme. The selectivity profile of **3D** against caspase-7, 8, and 9, and cathepsin-D was performed using Biomol kit (Enzo Life Sciences, Inc.). The enzyme activities were monitored (ex/em 355/460) as a time-course measurement of the increase in fluorescence signal from fluorescently labeled peptide substrate for 120 min at room temperature. Percent inhibition calculation was calculated using following equation:

$$\begin{array}{l} \text{Percent inhibition}=100\\ \;-[\left(\text{RFU in test wells}/\text{RFU in basal control wells}\right)\\ \;\times 100] \end{array}$$

### Mechanistic analysis of caspase-3 inhibition

Thirty microliters of caspase-3 (200 U) in assay buffer containing 30 μL of 20× NCE (the final compound concentration is approximately tenfold higher than respective IC_50s_). The mixture was incubated for 30 and 60 min at 30°C and then diluted 100-fold with assay buffer containing 10 μM AC-DEVD-AMC. The enzyme activity was measured at 30°C after 30 min. As a control, a mixture of 1× caspase-3 and 1× NCE was prepared and incubated for the same time duration before the addition of the substrate.

### Anti-apoptotic assay using Jurkat T-cells

The anti-apototic assay was done as described in [10]. Briefly, Jurkat (human T-cell leukemia) cell line (ATCC) was cultured in RPMI-1640 medium with l-glutamine and supplemented with 10% fetal bovine serum and antibiotic solution. Cells were grown at 37°C in 5% CO_2_ and 95% air environment at 100% humidity. Cells were plated in 45 μL/well of serum-free medium at 2.5 × 10^5^ cells/mL and incubated for 1 h before compound addition. Compound stock solutions were diluted with growth medium and added to the cells to obtain desired concentration. One percent DMSO was used for control wells (without the addition of compounds). Cells were incubated for 30 min before the addition of 5 μL of the apoptosis-inducer staurosporine (final concentration of 100 nM). After 24 or 48 h of incubation, resazurin reagent was added at 5.5 μL/well. Cells were further incubated for 120 min, and the fluorescence was measured using the Wallac Victor2V plate reader (PerkinElmer Life Sciences) at 531/589 nm ex/em wavelengths. The percent protection from induced apoptosis was calculated using the following equation:

$$\begin{array}{l} \text{Percent protection}=(\Delta Fl_{\text{t}}-\Delta Fl_{\text{s}})/(\Delta Fl_{\text{n}}-\Delta Fl_{\text{s}})\\ \;\times 100\text{,} \end{array}$$

where Δ*Fl* denotes the difference in resazurin fluorescence measured at point 0 and after 120-min incubation with the cells; subscripts t, s, and n are fluorescence in the presence of a test compound + staurosporine, staurosporine alone, and no staurosporine, respectively.

### ADME assays

#### Aqueous solubility

A high throughput kinetic solubility profiling was carried out by shake flask method in 96-well format at pH 7.4 and 5.4 with theoretical test concentration of 200 μM. After 16 h of incubation, the supernatant was subjected for analysis.

#### Permeability

PAMPA assay was carried out using PION kit at 10-μM test concentration according to the instructions provided by the manufacturer. Permeability assay using Caco-2 cell monolayer.

Briefly, Caco-2 cells (ATCC) were grown in DMEM supplemented with 10% fetal bovine serum, 1 mM non-essential amino acids, 1 mM sodium pyruvate, and gentamicin sulfate (50 μg/ml) to 70% to 80% confluency prior to seeding in 24-well plates loaded with polycarbonate Millicell inserts (12-mm diameter, 0.4 μm, 40,000 cells/insert; Millipore Co., MA, USA) at 37°C, 5% CO_2_ for 21 days. Cell monolayer integrity was assessed by measuring TEER. Drugs were applied at 10 μM in Hank's buffered salt solution to the apical or basal chamber, and transport assay was carried out for 2 h at 37°C in presence and absence of cyclosporin A. At the end of the assay, samples from both apical and basal chambers were collected for analysis, and the monolayer integrity was re-assessed by dye rejection using Lucifer yellow. Apparent permeability (*P*_app_) was calculated using the equation:

$$P_{\text{app}}\left(\text{cm}/\text{sec}\right)=\text{dQ}/\text{dt}\times 1/C_{o}\times 1/\text{A,}$$

where dQ/dt is the amount of drug transported within a given time period (μmol/sec); *C*_0_ is the initial concentration in the donor solution (μM); *A* is the surface area of insert filter membrane (cm^2^); and *t* is the incubation time (sec).

$$\text{Efflux ratio}\;\left(\text{ER}\right)=P_{\text{app }}{}_{\text{B to A}}/P_{\text{app A to B}}\text{,}$$

where *P*_app B to A_ is the *P*_app_ value measured in the B to A direction; *P*_app A to B_ is the *P*_app_ value measured in the A to B direction. Note that efflux ratios greater than 2 (or 3) are generally considered to be evidence for transport. Follow-up studies using inhibitors of drug transporters (e.g., cyclosporin A) was done to develop further evidence.

#### Microsomal stability

Mouse liver microsomes (in-house prepared) and pooled human liver microsomes (BD Gentest, CA, USA) were incubated with 1-μM test compounds at 37°C for 15 and 60 min using NADPH and UDPGA as co-factors. The reaction was stopped by the addition of cold acetonitrile, precipitated protein was removed, and the supernatants were analyzed.

#### Cytochrome P450 inhibition

Human recombinant CYP450 isozymes 1A2, 2 C9, 2 C19, 2D6, and 3A4 (BD Gentest) were incubated with 10 μM of test compound in buffer containing 0.1% DMSO for 10 min at 37°C, and the residual enzyme activity was measured using fluorogenic substrates as per BD Gentest protocol.

### Animals

Animal experimental procedures used in this study were approved by the Institutional Animal Ethical Committee based on the Committee for the Purpose of Control and Supervision on Experiments on Animals guidelines. Mice were used for the experiment after one-week acclimatization to standard laboratory conditions. Mice were fed with standard diet and water *ad libitum*.

### *In vivo* pharmacokinetic experiment in mice

Pharmacokinetic profiling was carried out using oral and intravenous route (IV) dosing in male NMRI mice. Three animals per route were administered test substance (1 mg/kg IV, 5 mg/kg PO) dissolved in vehicle containing 2% ethanol, 10% hydroxypropyl-β-cyclodextrin (HP-b-CD) and/or 1% Poloxamer 188 and quantity sufficient amount of 0.9% normal saline given at 10 ml/kg. The plasma samples were collected at different time points until 8 hr and were stored frozen at −80°C until analysis. Quantitative bio-analysis of the drugs in the plasma samples was done using an LC-MS/MS. Plasma samples were analyzed following protein precipitation of plasma with acetonitrile containing internal standards.

### Data fitting and statistical analysis

Curve fitting was performed with Prism 5.1 software (GraphPad Software Inc., San Diego, CA) using built-in equation describing corresponding data models. All the other data were mentioned as mean ± standard deviation of triplicates values. PK parameters were determined using WinNonlin 5.2 software.

## Results and discussion

### Biochemical screening

For screening of the compound library, the caspase-3 biochemical assay was first optimized. The fluorescence signal as well as signal to noise ratio was optimal at 10 U of caspase-3 and 10-μM substrate. Substrate exhibited Michaelis-Menten constant of 11.7 μM and was comparable to the published data [10]. The caspase-3 assay was validated using reference inhibitor (IDN6556) across all plates and different experiments [10-12].

**Figure 2:**
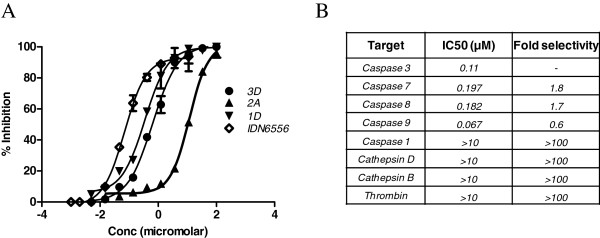
**Concentration-dependent inhibition and selectivity profile of NCEs.** (**A**) Concentration-dependent inhibition of caspase-3 with NCEs measured in presence of substrate (AC-DEVD-AMC) at 10 μM. The percentage of inhibition was calculated relative to the enzyme activity with no inhibitor present. (**B**) Selectivity profile of **3D** using Biomol kit (Enzo Life Sciences, Inc.). The enzyme activities were monitored (ex/em 355/460) as a time-course measurement of the increase in fluorescence signal from fluorescently labeled peptide substrate for 120 min at room temperature.

The primary screen of the 70 compounds at concentration of 1 and 10 μM revealed 22 hits (higher than 50% inhibition threshold at 10 μM) with dose-dependent inhibition (data not shown). The hits belong to all the four series, and most potent molecule is from indole chemotype, a chemotype previously reported to show good inhibition of cysteine proteases [4]. There was a clear trend in dose dependent inhibition of caspase-3 activity for all tested compounds from all four chemotypes (Figure 2A). It was observed that fluoromethylketone as a warhead is more potent than other substitutions like oxalamide. More bulky lipophilic groups substantially reduced the potency, and more polar and hydrophilic groups increased the potency. Electrophilic carbonyl from fluoromethylketone group might serve as sites of nucleophilic attack by cysteine thiolates in the caspase-3 catalytic domain. Few of these compounds were further characterized in the follow-up mechanistic assays, counterscreening assays against other proteases including caspase-1, 3, 7, 8, and 9, cathepsin-B and D, and thrombin.

### Time-dependent inhibition of caspase-3 activity

**Figure 3:**
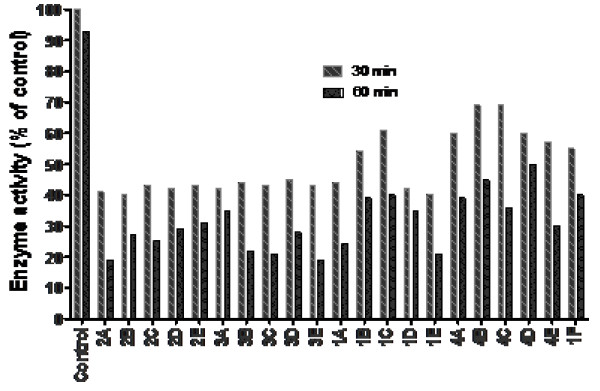
**Time-dependent inhibition of caspase-3 activity with NCEs.** Caspase-3 at a 20× concentration, as specified in the ‘Methods’ section, was pre-incubated with 20× NCE (the final compound concentration is approximately tenfold higher than respective IC_50s_). The mixture was pre-incubated for 30 and 60 min at 30°C and then diluted 100-folds with assay buffer containing 10-μM AC-DEVD-AMC. The enzyme activity was measured at 30°C after 30 min. As a control, a mixture of 1× caspase-3 and 1× NCE was prepared and incubated for the same time duration before addition of the substrate. The percentage inhibition was calculated relative to the activity of caspase-3, which went through all the procedures for concentrated or control samples but without the inhibitor. Note that compounds 1A to 3E are from indole, and 4A to 4F from oxalamide series.

Reversibility assessment of the caspase-3 activity with selected compounds was determined in the dilution experiment as described in the ‘Methods’ section. From the data shown in Figure 3, time-dependent inhibition of caspase-3 activity suggests irreversible interaction of compounds with caspase-3 enzyme and is similar to the reported caspase inhibitor IDN6556 [6,10,13].

### Counterscreen profile

The selectivity profile of selected compounds was assessed using a panel of proteases including cysteine proteases. The substrate concentration as well as other reaction conditions used for each assay is described in the ‘Methods’ section. The selectivity studies (refer to Figure 2B) indicated that compound **3D** was not selective towards caspases 7, 8, and 9 but good selectivity (>100-fold) over proteases including caspase-1, thrombin, cathepsin-B, and cathepsin D.

### Evaluation of anti-apoptotic activity

Protection against cellular apoptosis was determined using staurosporine as apoptotic inducer in Jurkat cells (human T-cell leukemia), as described in the ‘Methods’ section. Apoptosis in cells was measured as the suppression of resazurin reduction rate after 48-h incubation with staurosporine and in the presence and absence of compounds. Most of the compounds, with the exception of compound **3D**, showed minimal protection or no effect on staurosporine-induced apoptosis likely due to low permeability as observed in PAMPA assay (data not shown).

### Evaluation of compounds in *in vitro* ADME assay panel

The ADME profile for the selected set of compounds was determined using standard battery of assays including aqueous apparent solubility, microsomal metabolic stability, Caco-2 permeability, and drug-drug interaction studies using recombinant CYP450 enzymes. It is observed that the presence of polar group (such as acidic moiety) may help in good solubility and lack of transport of compound across the cell membrane in order to reach the target. Substitution of tetrafluorophenoxy showed interaction with efflux pump. Most of the compounds from fluoromethyl substitution on indole moiety and oxalamide showed relatively high microsomal stability compare to other substitutions. The optimal microsomal stability was found with substitution (Cl or OMe) on 5th position of indole with either difluoro and tetrafluorophenoxymethylketone substitution [see Table S1B in Additional file 1].

Inhibition of cytochrome P450 (CYP450) drug metabolizing enzymes may alter the metabolism of co-administered compounds, leading to a change in exposure and possible toxicity. Thus, the potential for the drug-drug interaction of NCEs was assessed by monitoring their impact on the metabolic activity of a panel of five major human CYP450 isoforms (3A4, 2 C9, 2 C19, 2D6, and 1A2) in the presence of a known fluorescent substrate for each of the isoform. CYP3A4 inhibition was determined in three assays using three distinct fluorescent substrates to account for the existence of two substrate binding sites. The results of these six assays are reported in Table S3 in Additional file 1. All the tested compounds showed no significant CYP450 inhibition under tested conditions, indicating no major alerts in drug-drug interactions.

### Pharmacokinetic profile of compounds in rodents

Compounds were administered to male NMRI mice either as 1 mg/kg intravenous bolus in 1% (*v*/*v*) DMA, 10% (*w*/*v*) HP-b-CD in normal saline, or orally by gavage at 5 mg/kg in 1% (*v*/*v*) DMA, 1% (*w*/*v*) Poloxamer 147, and 10% (*w*/*v*) HP-b-CD in saline. The relevant pharmacokinetic parameters reported in Table S4 in Additional file 1 indicate that the oral bioavailability of compound 3D (having tetrafluoro substitution) in this study was 90%, which was more when compared other compounds (such as series 1 and 4). A relatively high volume of distribution observed after IV dosing indicates that the compound gets distributed rapidly from the vascular compartment into the peripheral tissues. The plasma clearance was high (greater than hepatic blood flow) for most of the compounds tested except for compound **3D** with clearance value of 588 ml/h/kg (15% of hepatic blood flow).

Caspases play an important role in different pathophysiological conditions, and specific inhibition is expected to be therapeutically valuable. Towards identifying novel inhibitors of caspases with desirable selectivity, ADME and efficacy profile, we screened a focused library of compounds and identified inhibitors from four chemical series that are distinct from other known small molecule caspase-3 inhibitors. Among the hits identified with potency ranging from 0.11 to 50 μM IC_50_, compounds from two series that displayed potent caspase-3 inhibition were analyzed for reversibility. All the tested compounds from four series were found to be highly irreversible for their inhibition of caspase activity. Caspase-3 possess a total of seven cysteinyls, out of which three (excluding one in the active site) are readily accessible to water and, hence, to potential oxidation. It is well known in the literature that the compounds with warhead possess the irreversible type of caspase inhibition [13].

ADME properties including the permeability of the compounds with an objective of selecting the compounds with greater potential for cellular activity were analyzed. Although permeability of all the tested compounds were low, modest cellular activity suggested that compounds **3D** and **1D** were more potent than other compounds and may be because of marginally better permeability. Halogen (Cl) substitution on 5th position along with tetrafluorophenoxymethylketone substituent on right-hand side of molecule (as in compound **3D**) resulted in greater stability in liver microsomes [see Table S1A in Additional file 1] and higher interaction with efflux pump as indicated by higher efflux ratio in the Caco-2 permeability assays [see Table S3 in Additional file 1]. It also shows advantages in lowering the plasma clearance to about 10% of hepatic blood flow and increase in the oral bioavailability, which led to a good oral exposure. The interesting *in vitro* as well *in vivo* profile of compound ***3D*** supports further evaluation in efficacy models. However, such potential advantages need to be confirmed by additional characterization of compound **3D** in different pharmacological studies as well as *in vivo* toxicity evaluation.

## Conclusions

In summary, this study supports the hypothesis that caspase inhibition may protect cells from abnormal amount of apoptosis seen in a number of disease states. Continual treatment with a caspase inhibitor may preserve the loss of critical numbers of cells responsible for the development of caspase-mediated cell death. These results support further analysis of compound **3D** as a potential anti-apoptotic agent in pre-clinical models of apoptosis.

## Competing interests

The authors declare that they have no competing interests.

## Supplementary Material

Additional file 1 **Title.** Structure-activity relationship analysis and pharmacokinetic properties of NCEs. **Description:** Table S1 (A and B), structure-activity relationship analysis of hits around chemotype I, II and III derived from compound template; Table S2, structure-activity relationship analysis of hits around chemotype IV derived from compound template; Table S3, structure-activity relationship analysis of hits around chemotype I, III and IV derived from compound template; and S4, pharmacokinetic properties of NCEs. Click here for file
