# Supplementary material for: Discovery of indole tetrafluorophenoxymethylketone-based potent novel small molecule inhibitors of caspase-3
Source: Org Med Chem Lett. 2012 Jul 16;2:27. doi: 10.1186/2191-2858-2-27 (PMC3519673; doi:10.1186/2191-2858-2-27)
Supplement: Additional file 1 — Title. Structure-activity relationship analysis and pharmacokinetic properties of NCEs. Description: Table S1 (A and B), structure-activity relationship analysis of hits around chemotype I, II and III derived from compound template; Table S2, structure-activity relationship analysis of hits around chemotype IV derived from compound template; Table S3, structure-activity relationship analysis of hits around chemotype I, III and IV derived from compound template; and S4, pharmacokinetic properties of NCEs. [file 2191-2858-2-27-S1.doc]

# **Structure-activity relationship analysis and pharmacokinetic properties**

Table S1 (A and B): Structure-activity relationship analysis of hits around chemotype I, II and III derived from compound template

| **Cpd ID** | **Structures** | | **IC_50_ (µM)** | **% inhibition at 10 µM** | | | **% protection- 10 µM*** | **App solubility at pH7.4** | **% stable at 60 min** |
| --- | --- | --- | --- | --- | --- | --- | --- | --- | --- |
|  | **R** | **R^1^** | **Caspase-3** | **Thrombin** | **Caspase-1** | **Cathepsin-B** |  | **µM** | **MLM** |
| 1A | 5-Br | F | 0.58 | -9 | 4 | 4 | 5.5 | 181 | 67.5 |
| 1B | 2-Me | F | 1.23 | -6 | 6 | 4 | 15.3 | 176 | 98.5 |
| 1C | 5-OCH3 | F | 1.29 | -12 | 5 | 4 | 8.6 | 171 | 93.2 |
| 1D | 5-Cl | F | 0.35 | 21 | 8 | 6 | 31.7 | 200 | 71.7 |
| 1E | 5-F | F | 1.12 | -10 | 2 | 5 | 16.9 | 180 | 76 |
| 1F | t-butyl ester derivative of 1D | | 33.2 | -18 | 0 | 2 | 11.4 | 77.9 | 0 |
| 2A | 5-Br | 2,6-difluorophenox | 1.2 | -13 | 5 | 11 | 19.2 | 176 | 88 |
| 2B | 2-Me | 2,6-difluoro  phenoxy | 2.5 | -3 | 7 | 2 | 22.3 | 150 | 83.6 |

S1 (A)

*% protection against staurosporine-induced cell death at 100 nM. App, apparent; MLM, mouse liver microsomes.

| **Cpd ID**  S1 (B) | **Structures** | | **IC_50_ (µM)** | **% inhibition at 10 µM** | | | **% protection-10 µM*** | **App solubility at pH7.4** | **% stable at 60 min** |
| --- | --- | --- | --- | --- | --- | --- | --- | --- | --- |
|  | **R** | **R^1^** | **Caspase-3** | **Thrombin** | **Caspase-1** | **Cathepsin B** |  | **µM** | **MLM** |
| 2C | 5-OCH3 | 2,6-difluorophenoxy | 47.1 | -9 | 11 | 3 | 20.5 | 130 | 82 |
| 2D | 5-Cl | 2,6-difluorophenoxy | 20.8 | -11 | 15 | -3 | 21.5 | 118 | 64.6 |
| 2E | 5-F | 2,6-difluorophenoxy | 25.7 | -10 | 5 | 7 | 17.9 | 123 | 56 |
| 3A | 5-Br | 2,3,5,6-tetrafluorophenoxy | 0.988 | -14 | 3 | 9 | 6.8 | 171 | 15.3 |
| 3B | 2-CH3 | 2,3,5,6-tetrafluorophenoxy | 0.82 | 26 | 6 | 3 | 8.3 | 187 | 46.2 |
| 3C | 5-OCH3 | 2,3,5,6-tetrafluorophenoxy | 1.11 | -2 | 2 | 7 | 4.3 | 188 | 54.8 |
| 3D | 5-Cl | 2,3,5,6-tetrafluorophenoxy | 0.11 | 0 | 7 | 2 | 35.3 | 200 | 76.1 |
| 3E | 5-F | 2,3,5,6-tetrafluorophenoxy | 0.84 | -7 | 10 | 4 | 3.8 | 184 | 34.9 |

*% protection against staurosporine-induced cell death at 100 nM. App, apparent; MLM, mouse liver microsomes.

Table S2: Structure-activity relationship analysis of hits around chemotype IV derived from compound template

| **Cpd ID** |  |  | **IC_50_ (µM)** | **% inhibition at 10 µM** | | | **% protection-10 µM** | **App solubility at pH7.4** | **% stable at 60 min** |
| --- | --- | --- | --- | --- | --- | --- | --- | --- | --- |
|  | **R** | **R1** | **Caspase-3** | **Thrombin** | **Caspase-1** | **Cathepsin-B** |  |  |  |
|  |  |  |  |  |  |  |  | **µM** | **MLM** |
| 4A | 4-F |  | 14.2 | -11 | 5 | 0 | 18.1 | 199 | 92.6 |
| 4B | 2-OCH_3_ |  | 8.4 | -10 | 4 | -2 | 14.9 | 200 | 72.3 |
| 4C | 3-CH_3_ |  | 25.2 | -11 | 6 | -3 | 12.5 | 200 | 84.3 |
| 4D | 4-CH_3_ | F | 9.67 | -7 | 3 | -5 | 1.3 | 139 | 75.7 |
| 4E | 4-F | F | 3.47 | -15 | 4 | -2 | 11.3 | 147 | 73.6 |
| IDN  6656 | - | - | 0.035 | 7 | 59 | 0 | 73 | 145 | 61 |

*% protection against staurosporine-induced cell death at 100 nM. App, apparent; MLM, mouse liver microsomes.

Table S3: Structure-activity relationship analysis of hits around chemotype I, III and IV derived from compound template

| **Cpd ID** | **R1** | **R2** | **Caco-2** | **CYP inhibition at 10 µM**  **rHCYP450** | | | |
| --- | --- | --- | --- | --- | --- | --- | --- |
|  |  |  | **P_app_ (cm/sec) and**  **efflux ratio** | **1A2** | **2C9,19** | **2D6** | **3A4** |
| 1A | Br | F | <2E-06  0.86 | 12 | -6/16 | -1 | 1/10/15 |
| 1D | Cl | F | <2E-06  1.1 | 11 | -10/7 | -4 | 2/0/15 |
| 1E | F | F | Not done | 13 | -10/1 | -3 | -5/0/15 |
| 3D | Cl |  | <2E-06  63.9 | 7 | -1/29 | 2 | 10/-4/10 |
| 3E | F |  | Not done | 12 | -1/3 | 0 | 17/-2/12 |
| 4E |  | | <2E-06  2.1 | 17 | -9/8 | 3 | -1/0/14 |

|  | **K_el_** | **Beta t_1/2_** | **AUC _(0-inf)_ iv** | **V_d_** | **Cl** | **AUC _(0-inf)_ po** | **T_max_** | **C_max_** | **F** |
| --- | --- | --- | --- | --- | --- | --- | --- | --- | --- |
| **Cpd ID** | **(hr-1)** | **(hr)** | **(hr ng/ml)** | **(mL/kg)** | **(mL/hr/kg)** | **(hr ng/ml)** | **hr** | **ng/ml** | **%** |
| 3D | 0.3 | 2.1 | 1698.8 | 1790 | 588 | 7648.8 | 0.5 | 7733 | 90.1 |
| 1A | 1.6 | 0.4 | 91.1 | 6700 | 10980 | 264.7 | 0.5 | 387.5 | 58.1 |
| 1D | 1.25 | 0.56 | 182.5 | 4390 | 5478 | 329.1 | 0.25 | 542 | 36.1 |
| 4E | 1.36 | 0.51 | 93.6 | 7880 | 10680 | 259.3 | 0.5 | 389.3 | 55.4 |
| IDN6556 | 0.7 | 0.9 | 110.7 | 11770 | 9030 | 99.1 | 0.5 | 113 | 17.9 |

Table S4: Pharmacokinetic properties of NCEs
